# Effects of dietary digestible lysine levels in breeding Japanese quails on productive and reproductive performance, egg quality, blood metabolites and immune responses

**DOI:** 10.1002/vms3.70038

**Published:** 2024-10-05

**Authors:** Mohammad Amin Omary, Heydar Zarghi, Ahmad Hassanabadi

**Affiliations:** ^1^ Department of Animal Science, Faculty of Agriculture Ferdowsi University of Mashhad Mashhad Iran

**Keywords:** breeding Japanese quail, digestible lysine, requirement, regression model

## Abstract

**Background:**

The vegetable‐based diet alone does not provide the lysine (Lys) needed to maximize poultry productive performance.

**Objectives:**

This experiment aimed to evaluate the effects of dietary digestible Lys (dLys) level on productive and reproductive performance, egg quality, blood metabolites and immune responses in breeding Japanese quails (*Coturnix japonica*).

**Methods:**

The experiment was conducted in a completely randomized design with 6 treatments, 5 replicates and 15 (12 females and 3 meals) 10‐week‐old breeding Japanese quails each. A basal diet was formulated to meet nutritional requirements of breeding quails except dLys. The basal diet was supplemented with graded (+0.82 g/kg) levels of l‐Lys‐HCl, corresponding to dietary dLys levels of 0.690%, 0.755%, 0.820%, 0.885%, 0.950% and 1.015%. The experiment lasted for 12 weeks, which was divided into 3‐4‐week periods.

**Results:**

Significant differences were observed for egg production (EP), egg mass (EM) and feed efficiency (FE) in response to increasing dietary dLys concentration with quadratic trends. The highest traits were observed in the birds fed with a diet containing 0.885% dLys. However, feed intake, egg quality, reproductive performance, blood metabolites and immune responses against sheep red blood cell inoculation were not significantly affected by increasing dietary dLys concentrations. The dLys requirements during 11–14, 15–18, 19–22 and 11–22 (overall) weeks of age for optimal EP, EM and FE, based on the quadratic broken‐line regression analysis, were estimated 272, 265, 250 and 266; 293, 285, 264 and 279; and 303, 294, 281 and 293 mg/bird/day, respectively.

**Conclusions:**

The dLys requirements vary depending on the EP phase and the trait being optimized. The estimated dLys requirement for FE was higher than those for EP and EM. During the peak stage of the first laying cycle, the dietary dLys level of 0.932% and a daily intake of 303 mg dLys/bird are sufficient for optimal performance.

## Graphical abstract

In the breeding Japanese quail, the digestible lysine requirements vary depending on traits are considered for optimization. The digestible lysine requirement estimated for feed efficiency was higher than those for egg production. During the peak stage of first laying cycle, a daily intake of 293 mg digestible lysine per bird is adequate for optimized performance.

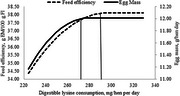

## 1 INTRODUCTION

In avian species, lysine (Lys) is an indispensable amino acid (Schutte & Smink, 1998). It is required to maintain the normal physiological functions, such as protein synthesis (Ribeiro et al., 2003), lipid metabolism (Fouad et al., 2018; Fouad & El‐Senousey, 2014; Robbins et al., 2006) and carnitine synthesis, which is an essential limiting step in the fatty acids transportation to mitochondrial β‐oxidation, bone matrix formation (Fischer et al., 2008; Lima et al., 2016; Tatiane da Silva et al., 2021), satisfactory immunity (Taghinejad‐Roudbaneh et al., 2011), productive (Lima et al., 2016; Pinto et al., 2003; Shim & Lee, 1984, 1993) and reproductive performance (Fouad et al., 2018; Shim & Lee, 1985).

The vegetable feedstuff‐based diets do not provide the Lys amounts needed to maximize protein deposition, egg size and mass. Therefore, the use of Lys from synthetic sources is necessary (Tatiane da Silva et al., 2021). Data regarding the effects of dietary Lys supplementation on avian species, including broilers, turkeys and laying hens, are available (Fouad et al., 2018; Shim & Lee, 1985), but information about breeding Japanese quails is scanty (Kaur et al., 2008). Additionally, there are contradictory results in the estimation of their amino acid requirements. Therefore, breeding quail's diets are formulated in accordance with the recommendations for commercial laying quails (Carvalho et al., 2022). Breeding quails may have differences in egg production (EP), egg weight (EW) and egg mass (EM) when compared to commercial laying quails, which drives the need for further research in the amino acid requirements for these birds (Rostagno et al., 2011, 2017). Dietary Lys concentration for Japanese quail during the laying phase was suggested at 0.79% (Shrivastav et al., 1990), 0.97%–1.0% (Shim & Lee, 1984, 1993), 1.0% (NRC, 1994), 1.0%–1.1% (Shim & Lee, 1985) and 1.17%–1.23% (Rostagno et al., 2011). Therefore, the objective of the current study was to determine the effects of different dietary levels of digestible Lys (dLys) during the early stages of laying period on productive and reproductive performance, egg quality, blood metabolites, concentration of liver function enzymes and immune responses in breeding Japanese quails (*Coturnix japonica*). Additionally, to determine the dLys requirement from the optimized point obtained for productive and reproductive performance.

## 2 MATERIALS AND METHODS

### 2.1 Feedstuff's analysis

Prior to the trial, crude protein (CP), total and digestible amino acids of feed ingredients were measured by using near infra‐red analysis through Evonik Co. (Evonik Nutrition & Care GmbH) agent in Tehran, Iran. These values were used in a least‐cost equation for the formulation of the experimental diets.

### 2.2 Birds, housing, experimental design and diets

Japanese quails, which had been selected for meat and EP in the Pishgaman Quail Breeding Cooperative Company of Khorasan, Mashhad, Iran (Registration Number: 70675), were reared with commercial starter and grower diets in floor area until 6 weeks of age. The birds were moved from the floor to laying battery cages in an environmentally controlled room. The birds were kept in galvanized wire cages (50 × 40 × 40 cm^3^ in size; L × W × H), corresponding to 133 cm^2^ per bird, and equipped with trough feeders placed in the front section of each cage with corresponding nipple drinkers. They were fed with a commercial breeder diet until maximum EP and nearly maximal EP and egg size were achieved at 10 weeks of age (laying peak). At this age (9–10 weeks), the live body weight (LBW) (334 ± 7.44 g/bird), weight gain (0.05 g/bird/day), EM (10.5 ± 0.47 g/bird/day) and feed consumption (30 ± 1.56 g/bird/day) were determined. The breeding quail nutritional requirements, including daily metabolizable energy (ME) and dLys requirement for maintenance, weight gain and EM, were calculated by the equations (Rostagno et al., 2011), and the indispensable amino acid requirements were determined using amino acids/Lys ratio recommended in Brazilian Tables for Poultry and Swine (Rostagno et al., 2011, 2017).

A total of 450 (360 females and 90 males) healthy mature Japanese quails with almost similar LBW and average EP were randomly divided into 6 treatment groups (60 females and 15 males/group). Each group was subdivided into 5 replicates with 12 females and 3 males. A basal diet was formulated based on the ingredient analysis, and birds feed consumption ability to meet the calculated nutritional requirements of Japanese quails except for dLys. The experimental diets were prepared in a way that a batch of non‐Lys supplemented basal diet (0.69% dLys concentration) was created and then divided into six equal parts. Then, l‐Lys‐HCl (ME, 4120 kcal/kg; CP, 93.4%; and Lys, 78.8% with 100% digestibility; Evonik Degussa GmbH) was added at the rate of 0.00, 0.82, 1.64, 2.46, 3.28 and 4.1 g/kg to the top of each division at the expense of filler (corn starch) and mixed to provide six experimental diets with 0.690%, 0.755%, 0.820%, 0.885%, 0.950% and 1.015% dLys concentration (Table 1). From 11 to 22 weeks of age, the birds were fed with six experimental diets, and the whole experimental period was divided into three consecutive periods of 28‐day for data collection. The house temperature was maintained at 21 ± 2°C for the whole period. Incandescent lamps were used to supply a lighting schedule of 16L:8D h with a light intensity of four watts per square meter of room floor throughout the experiment. The light supply was controlled by a timer, allowing the lights to be turned on and off at night and in the early hour of the morning, following the procedure adopted in commercial poultry farms.

**TABLE 1:** Ingredients and nutrients composition of the basal diet.^a^

Items	Basal diet
**Ingredient, g/kg as‐fed basis**	
Corn (ME = 3363, CP = 7.93)	624.3
Wheat (ME = 3213, CP = 12.74)	47.2
Soybean meal (ME = 2536, CP = 40.94)	240.4
Limestone	61.4
Dicalcium phosphate	7.6
Common salt	2.7
Vitamin premix ^b^	2.5
Mineral premix ^c^	2.5
l‐tryptophan	0.4
dl‐ methionine	3.4
l‐arginine	2.0
l‐threonine	0.9
l‐valine	0.6
Corn starch	4.1
**Determined nutrients composition** ^d^ **, as‐fed basis**	
Metabolizable energy, kcal/kg	2900
Crude protein, %	16.50
Calcium, %	2.56
Available phosphorus, %	0.27
Sodium, %	0.13
Digestible methionine, %	0.56
Digestible sulphur amino acids (methionine + cystine), %	0.80
Digestible lysine, %	0.69
Digestible threonine, %	0.59
Digestible valine, %	0.73
Digestible tryptophan, %	0.20
Digestible arginine, %	1.12
Acid linoleic, %	1.67

Abbreviations: CP, crude protein; ME, metabolizable energy.

^a^ The experimental diets were provided in a way that a batch of basal diet (lowest digestible lysine concentration) was made and then divided into six equal portions; the l‐lysine‐HCl (metabolizable energy = 4120 kcal/kg, crude protein = 93.4% and lysine = 78.8%, Evonik Degussa GmbH) was added at the rate of 0.00, 0.82, 1.64, 2.46, 3.28 and 4.10 g/kg to the top of each portion at the expense of filler (corn starch) and mixed to provide six experimental diets with 0.690%, 0.755%, 0.820%, 0.885%, 0.950% and 1.015% digestible lysine levels, respectively.

^b^ Vitamin premix supplied the following per kilogram of diet. Vitamin A (all‐trans‐retinol), 4400 IU; Vitamin D3 (cholecalciferol), 1000 IU; Vitamin E (a‐tocopherol), 11 IU; Vitamin K3 (menadione), 2.33 mg; Vitamin B1 (thia‐min), 2.97 mg; Vitamin B2 (riboflavin), 4.4 mg; Vitamin B3 (niacin), 22 mg; Vitamin B5 (pantothenic acid), 10 mg; Vitamin B6 (pyridoxine), 4.45 mg; Vitamin B9 (folic acid), 1.9 mg; Vitamin B12 (cyanocobalamin), 0.011 mg; Vitamin H2 (biotin), 0.18 mg; choline chloride, 487.5 mg and antioxidant, 1.0 mg.

^c^ Mineral premix supplied the following per kilogram of diet. Zn (zinc oxide), 75 mg; Mn (manganese oxide), 75 mg; Fe (iron sulphate), 75 mg; Cu (copper sulphate), 5 mg; I (ethylene diamine di‐hydroiodide), 0.76 mg; Se (Sodium Selenite), 0.1 mg and choline chloride, 474 mg.

^d^ The determined ingredient analysis was used to calculate nutrient composition (crude protein, calcium and sodium were measured by the AOAC (2002) methods; metabolizable energy, digestible amino acids and available phosphorus were measured by using the near infra‐red analysis; methionine + cystine estimated with separate calibration equation.

### 2.3 Data collection and sampling

#### 2.3.1 Productive traits

The quails were weighed in groups at the beginning and end of the experiment. The LBW changes were calculated as the difference between the initial and final LBW. The number and weight of all eggs produced in each experimental unit were recorded daily. Mortality was recorded as it occurred. The feed consumption of birds in each pen was calculated by subtracting the amount of feed remaining at the end from the total feed given during each 28‐day period and adjusted for mortality. Productive performance traits, such as feed intake (FI), EP, EW, EM and feed efficiency (FE), were calculated and compiled during periods of 11–14, 15–18 and 19–22 weeks of age, and in the whole experimental period (11–22 weeks of age). The daily ME, CP and dLys consumption for each period (d28) were calculated using FI information and diet nutrient composition (Table 1).

#### 2.3.2 Egg quality

At the end of each 4‐week period, 4 eggs/replicates (20/treatment) were randomly selected from the laid eggs and sent to the Egg Quality Lab within 6 h of collection. The eggs were weighed by a digital electronic scale (0.001 g, Model GF 400, A&D Weighing Co. Ltd.). The weight of each egg was determined when immersed in distilled water to calculate egg specific gravity (SG) by Archimedes method using the following formula (Hempe et al., 1988):

$$\begin{array}{ccc} & & \mathrm{Egg}\;\mathrm{specific}\;\mathrm{gravity},\;g/\;cm^{3}\\ & & \;=\;\frac{\mathrm{Egg}\;\mathrm{weight}}{\mathrm{Egg}\;\mathrm{weight}-\mathrm{Egg}\;\mathrm{weight}\;\mathrm{immersed}\;\mathrm{in}\;\mathrm{water}\;} \end{array}$$

The egg length and width were determined by passing the width or length of eggs through the digital Caliper (0.05 mm, Model 1116‐150, Insize Co. Ltd.) to calculate the egg shape index by the following formula (Altuntaş & Şekeroğlu, 2008):

$$\mathrm{Shape}\;\mathrm{index}\;=\frac{\mathrm{Egg}\;\mathrm{width}}{\mathrm{Egg}\;\mathrm{length}}\times 100$$

To measure internal and external egg quality characteristics, the egg was carefully broken onto a glass plate (35 × 25 cm^2^). Haugh unit was calculated based on the following formula (Haugh, 1937):

$$\begin{array}{ccc} & & \mathrm{Haugh}\;\mathrm{unit}=100\\ & & \;\times \;\log[\mathrm{Albumen}\;\mathrm{height}\;\left(\mathrm{mm}\right)+7.57-1.7\times \mathrm{Egg}\;\mathrm{weight}\;{\left(g\right)}^{0.37}] \end{array}$$

Yolk and albumen were separated by a commercial hand‐held egg separator. Cloth napkin was applied to eradicate the adhering of albumen remains from the yolk, and then it was weighed. The eggshell was rinsed with distilled water, dried for 48 h and weighed. The albumen weight was calculated by subtracting yolk + shell weights from the whole EW. Eggshell thickness was measured employing a micrometre device (0.001‐mm, Model 293‐240, Mitutoyo Co., Ltd.) at three disparate sites, including air sack, equator and sharp end, that were averaged to determine eggshell thickness (Hossaninejad et al., 2021).

#### 2.3.3 Egg composition

At the end of the experimental period, 10 eggs/replicates were randomly selected for measuring egg composition. After breaking, yolk and albumen were separated as previously explained. Yolks and albumen were pooled in separate containers and homogenized to make composite samples. The solid content of yolk and albumen was determined by drying in an electric oven at 75°C for 72 h. The extract content of yolk was determined by the extraction method. Nitrogen content of yolk and albumen was analysed by using a nitrogen analyser. A factor of 6.25 was used to calculate CP content from the nitrogen values (AOAC, 2002).

#### 2.3.4 Fertility and hatchability

At the latest week of the experiment, 50 eggs from each replicate (250/treatment) were collected and incubated. An incubating temperature of 37.5°C and a relative humidity of 60% wet bulb reading of 30°C were applied until the 14th day of incubation. The eggs were turned 90° in the longitudinal axis every 2–4 h. On Day 14 of incubation, the eggs were candled and any cracked, infertile and dead embryo eggs were removed. Then the eggs were transferred to hatching trays, and the turning was stopped. A separate hatcher was used with a temperature of 37.2°C and a relative humidity of 70% wet bulb reading of 32.2°C. The hatched chicks were removed on the 17th or 18th day of incubation, counted and weighted. Then non‐hatched eggs were broken to calculate the fertility and hatchability percentages. The hatchability was expressed in two ways of hatched chicks from fertile eggs and hatched chicks from total egg sets (Alagawany et al., 2014). Fertility and hatchability percentages were calculated as follows:

$$\mathrm{Fertility}\;\%\;=\frac{\mathrm{Number}\;\mathrm{of}\;\mathrm{fertile}\;\mathrm{eggs}}{\mathrm{Total}\;\mathrm{eggs}\;\mathrm{set}}\;\times 100$$

$$\begin{array}{ccc} & & \mathrm{Hatchability}\;\%\;\left(\mathrm{from}\;\mathrm{total}\;\mathrm{fertile}\;\mathrm{eggs}\right)\\ & & \;=\;\frac{\mathrm{Number}\;\mathrm{of}\;\mathrm{hatched}\;\mathrm{chicks}}{\mathrm{Total}\;\mathrm{number}\;\mathrm{of}\;\mathrm{fertile}\;\mathrm{eggs}}\times 100 \end{array}$$

$$\begin{array}{ccc} & & \mathrm{Hatchability}\;\%\;\left(\mathrm{from}\;\mathrm{total}\;\mathrm{set}\;\mathrm{eggs}\right)\\ & & \;=\;\frac{\mathrm{Number}\;\mathrm{of}\;\mathrm{hatched}\;\mathrm{chicks}}{\mathrm{Total}\;\mathrm{eggs}\;\mathrm{set}}\times 100 \end{array}$$

#### 2.3.5 Humoral immune response

Humoral immune response was evaluated by haemagglutination (HA) antibody titre estimation. A 5% suspension of sheep red blood cell (SRBC) in phosphate buffered saline was prepared and stored under refrigeration at 4°C until use. At the end of 20 and 21 weeks of experimental period, five female birds per treatment were selected to study the primary antibody response and 0.5 mL of SRBC suspension was injected intramuscularly to each bird. At Day 7 after SRBC injection, 1 mL of blood was taken from the wing vein. After allowing for the completion of clotting, blood samples were centrifuged at 1900 *g* for 5 min at 4°C to extract serum and frozen (−20°C) until analysed for antibody titres to SRBC. The antibody titre was determined by the HA test, and titres were expressed as log_2_ (Hossaninejad et al., 2021).

#### 2.3.6 Blood collection and analysis

At the end of the experiment, one bird from each replicate (five/treatment) was randomly selected, and the blood sample was taken from the brachial vein of each bird into non‐heparinized tubes. After allowing for the completion of clotting, blood samples were centrifuged at 1900 *g* for 5 min at 4°C to extract serum (Hossaninejad et al., 2021). Blood serum metabolites, including triglyceride, total cholesterol (Chol), high‐density lipoprotein Chol, total protein, creatinine, uric acid (UA), alanine aminotransferase, aspartate aminotransferase and alkaline phosphatase (ALP) concentrations, were measured. To measure blood metabolites, the kits of Pars Azmoon Company and a multi‐test automatic random‐access system auto‐analyser (Cobas Bio, Roche Basel) were used.

### 2.4 Statistical analysis

The experiment was done in a completely randomized design. All data were analysed for normality by applying SAS 9.4 computer software through univariate plan normal procedure (SAS, 2014). The data were analysed by regression procedure for linear and quadratic responses to dietary dLys concentrations. The dLys requirements for optimal responses were determined by utilization of analysed daily dLys intakes using non‐linear (NLIN) procedures, through linear and quadratic broken‐line regression analysis (Robbins et al., 2006). The iterative procedure makes repeated estimates for coefficients and minimizes residual error until the best‐fit line is achieved. The configuration model was selected based on the highest adjusted coefficient of determination (adj. *R* ^2^), and the lowest root means square error and the lowest Akaike's information criterion values were calculated (Hossaninejad et al., 2021). Finally, the quadratic broken‐line regression analysis was used as follows:

$$Y=L+U\;\times {\left(R-X\right)}^{2}\times I$$

where *Y* is the dependent variable, *L* is the theoretical maximum, *R* is the requirement, *X* is the independent variable, *I* is 1 (if *X* < *R*) or *I* is 0 (if *X* > *R*) and *U* is the rate constant.

## 3 RESULTS

### 3.1 Feed and nutrients intake

The effect of dietary dLys levels on FI, ME, CP and dLys intake of breeding Japanese quails during 11–14, 15–18 and 19–22 weeks of age periods and the whole experimental period (11–22 weeks of age) are shown in Table 2. The FI, ME and CP intake was not significantly affected by the increasing levels of dietary dLys (*p* > 0.05). Under the conditions of the current study, the birds had an average daily FI of 32.49 ± 1.05 g, 94.5 ± 3.06 kcal of ME and 5.36 ± 0.17 g of CP per bird during the whole experimental period. Based on the feed consumption and dietary amino acid analysis results, increasing dietary dLys levels from 0.69% to 1.015% leads to a step‐by‐step increase in daily dLys intake. The dLys consumptions were linearly increased (*p* < 0.001) from 234 to 335, 233 to 337, 222 to 297 and 230 to 323 mg/bird/day during 11–14, 15–18 and 19–22 weeks of age and the whole experimental period, respectively.

**TABLE 2:** Effect of diet digestible lysine (dLys) concentration on feed consumption, metabolizable energy (ME), crude protein (CP), analysed dLys intake of breeding Japanese quail during periods 11–14, 15–18 and 19–22 weeks of age and the whole experiment (overall: 11–22 weeks).

	Feed consumption, g/bird/day				ME intake, kcal/b/d				CP intake, g/bird/day				Analysed dLys intake, mg/bird/day			
Analysed dLys concentration, %	11–14 weeks	15–18 weeks	19–22 weeks	Overall	11–14 weeks	15–18 weeks	19–22 weeks	Overall	11–14 weeks	15–18 weeks	19–22 weeks	Overall	11–14 weeks	15–18 weeks	19–22 weeks	Overall
0.690	33.43	33.82	30.37	32.51	98.58	98.18	93.62	96.79	5.59	5.57	5.31	5.49	234	233	222	230
0.755	32.98	32.73	30.71	32.28	97.69	95.98	92.01	95.23	5.54	5.44	5.22	5.40	253	249	239	247
0.820	33.95	33.13	30.71	32.47	99.19	96.46	89.36	95.00	5.62	5.47	5.07	5.39	280	272	252	268
0.885	33.41	32.96	30.71	32.35	98.15	98.29	89.39	95.28	5.57	5.57	5.07	5.40	298	299	272	290
0.950	33.09	32.79	29.92	30.98	97.72	92.83	86.57	92.38	5.54	5.26	4.91	5.24	319	303	283	302
1.015	32.25	31.02	28.56	30.64	96.00	96.64	85.20	92.61	5.44	5.48	4.83	5.25	335	337	297	323
SEM	0.327	0.511	0.746	0.403	0.919	1.517	2.178	1.161	0.054	0.084	0.123	0.066	2.809	4.540	6.667	3.625
Dietary dLys levels response, *p*‐value																
Linear	0.250	0.599	0.840	0.958	0.217	0.672	0.824	0.961	0.244	0.588	0.849	0.964	0.004	0.001	0.001	0.001
Quadratic	0.214	0.640	0.978	0.917	0.183	0.713	0.961	0.917	0.208	0.627	0.986	0.912	0.145	0.727	0.764	0.661

*Note*: Data are means of 5 replications of 15 (12 female + 3 male) birds each.

Abbreviations: CP, crude protein; ME, metabolizable energy.

### 3.2 Live body weight change

The average LBW of quails at the beginning of the experiment was 348 ± 8.4 g/bird. Dietary treatments had no significant influence (*p* > 0.05) on quails LBW during the experiment. At the end of the experiment, the birds average LBW reached to 339 ± 20 (Table 3).

**TABLE 3:** Effect of diet digestible lysine (dLys) concentration on productive performance and body weight changes of breeding Japanese quail during periods 11–14, 15–18 and 19–22 weeks of age and the whole experiment (overall: 11–22 weeks).

	Egg production, egg/100 hen day				Egg mass, g egg/hen day				Feed efficiency, (g egg mass/100 g feed intake)				Live body weight, (g)		
Analysed dLys concentration, %	11–14 weeks	15–18 weeks	19–22 weeks	Overall	11–14 weeks	15–18 weeks	19–22 weeks	Overall	11–14 weeks	15–18 weeks	19–22 weeks	Overall	Initial	Final	Change
0.690	84.30	86.44	76.58	82.47	11.66	11.88	10.69	11.36	34.86	35.13	35.21	34.94	354	348	−6
0.755	85.26	88.36	83.49	85.03	11.81	11.74	11.49	11.68	35.80	35.88	37.42	36.19	346	341	−5
0.820	88.43	90.51	83.56	87.42	12.37	12.78	11.48	12.20	36.42	38.58	37.38	37.57	347	335	−12
0.885	89.17	91.05	86.83	88.57	12.32	12.88	12.05	12.48	36.87	39.06	39.24	38.58	351	339	−12
0.950	88.10	90.06	82.11	84.46	12.19	12.83	11.45	11.73	36.85	39.12	38.27	37.87	348	325	−23
1.015	87.37	89.26	82.05	84.96	11.85	11.67	10.96	11.58	36.74	37.63	38.37	37.79	342	348	6
SEM	1.004	1.438	1.602	1.069	0.183	0.256	0.278	0.211	0.116	0.247	0.457	0.263	3.336	8.363	8.393
Dietary dLys levels response, *p*‐value															
Linear	0.012	0.019	0.005	0.003	0.012	0.051	0.011	0.009	0.066	0.008	0.012	0.004	0.959	0.153	0.157
Quadratic	0.018	0.024	0.006	0.004	0.014	0.045	0.010	0.008	0.084	0.007	0.016	0.005	0.883	0.161	0.155

*Note*: Data are means of 5 replications of 15 (12 females + 3 males) birds each.

### 3.3 Productive performance

The effects of dietary dLys levels on productive performance traits are shown in Table 3. These results demonstrated that l‐Lys‐HCl supplementation had a significant effect with quadratic trends on EP performance (*p* < 0.05). The results showed that the birds fed with supplemented diet by 1.64 g l‐Lys‐HCl/kg of diet (contained 0.885% dig Lys) showed the highest EP, EM and FE. During the whole experimental period, the EP, EM and FE were improved by 7.40%, 8.10% and 8.85% when the birds fed with a diet containing 0.885% dLys in compare to the birds fed with a non‐supplemented diet. However, increasing the dietary dLys concentrations to 0.95% and 1.015% led to a decrease in the afore‐mentioned performance traits. The same discernible improvements were observed during the first, second and third experimental periods.

### 3.4 Egg weight, quality and composition

The effect of dietary dLys concentration on egg quality traits is shown in Table 4 (EW and egg compounds relative weight), Table 5 (egg shape index, Haugh unit, SG and eggshell thickness) and Table 6 (egg composition). As indicated in these tables, all egg quality and composition traits were not significantly affected by l‐Lys‐HCl supplementation during any experimental period and throughout the experiment (*p* > 0.05).

**TABLE 4:** Effect of diet digestible lysine (dLys) concentration on egg qualitative traits of breeding Japanese quail during periods 11–14, 15–18 and 19–22 weeks of age and the whole experiment (overall: 11–22 weeks).

Analysed dLys lysine concentration, %	Egg weight, g/egg				Albumen (g/100 g egg)				Yolk (g/100 g egg)				Shell (g/100 g egg)			
	11–14 weeks	15–18 weeks	19–22 weeks	Overall	11–14 weeks	15–18 weeks	19–22 weeks	Overall	11–14 weeks	15–18 weeks	19–22 weeks	Overall	11–14 weeks	15–18 weeks	19–22 weeks	Overall
0.690	13.83	13.75	13.99	13.77	62.86	60.79	60.85	61.50	28.75	30.89	30.68	30.11	8.39	8.32	8.47	8.39
0.755	13.84	13.29	13.77	13.74	60.21	61.50	61.19	60.97	31.15	30.16	30.61	30.64	8.63	8.34	8.20	8.39
0.820	13.99	14.13	13.74	13.96	61.62	60.61	61.01	61.08	30.01	31.31	30.38	30.57	8.37	8.08	8.61	8.35
0.885	13.81	14.14	13.89	14.09	62.10	63.73	61.88	62.57	28.96	28.06	30.21	29.08	8.94	8.21	7.91	8.35
0.950	13.84	14.24	13.94	13.89	59.19	62.91	62.26	61.45	31.65	28.85	29.54	30.01	9.16	8.24	8.20	8.53
1.015	13.57	13.08	13.36	13.64	60.28	61.23	57.84	59.79	31.25	30.79	33.79	31.95	8.47	7.97	8.36	8.27
SEM	0.139	0.124	0.159	0.125	0.884	0.941	1.149	0.748	0.796	0.905	1.056	0.693	0.212	0.229	0.264	0.147
Dietary dLys levels response, *p*‐value																
Linear	0.315	0.171	0.521	0.263	0.736	0.212	0.078	0.191	0.983	0.196	0.079	0.149	0.197	0.918	0.504	0.742
Quadratic	0.282	0.133	0.433	0.211	0.813	0.233	0.068	0.176	0.942	0.213	0.068	0.135	0.220	0.877	0.522	0.734

*Note*: Data are means of five replications of four eggs each that measured at the end of each period.

**TABLE 5:** Effect of digestible lysine (dLys) concentration on egg qualitative traits of breeding Japanese quail during periods 11–14, 15–18 and 19–22 weeks of age and the whole experiment (overall: 11–22 weeks).

	Egg shape index (%)				Haugh unit				Specific gravity (g/cm^3^)				Shell thickness (µm)			
Analysed dLys concentration, %	11–14 weeks	15–18 weeks	19–22 weeks	Overall	11–14 weeks	15–18 weeks	19–22 weeks	Overall	11–14 weeks	15–18 weeks	19–22 weeks	Overall	11–14 weeks	15–18 weeks	19–22 weeks	Overall
0.690	76.64	74.35	78.11	76.37	80.81	88.52	87.83	85.72	1.067	1.068	1.063	1.066	255	260	253	253
0.755	78.09	76.88	77.03	77.34	82.62	86.61	86.59	85.27	1.064	1.068	1.064	1.065	247	273	260	260
0.820	77.93	76.61	77.19	77.24	85.41	86.73	87.23	86.46	1.066	1.065	1.065	1.065	255	247	251	251
0.885	78.34	78.99	78.97	78.77	81.88	83.11	85.25	83.41	1.069	1.069	1.061	1.066	268	259	263	263
0.950	78.82	77.27	77.38	77.82	83.34	88.18	88.57	86.70	1.070	1.067	1.062	1.066	269	253	266	266
1.015	78.25	77.53	76.68	77.49	83.60	88.98	88.20	86.93	1.067	1.063	1.066	1.066	259	253	256	256
SEM	0.912	0.799	1.329	0.708	2.154	1.601	1.247	0.984	0.002	0.003	0.003	0.002	8.342	7.113	6.031	6.031
Dietary dLys levels response, *p*‐value																
Linear	0.342	0.131	0.727	0.141	0.518	0.154	0.253	0.290	0.566	0.640	0.461	0.971	0.131	0.250	0.472	0.472
Quadratic	0.380	0.143	0.711	0.161	0.541	0.152	0.242	0.271	0.613	0.606	0.451	0.973	0.232	0.839	0.501	0.501

*Note*: Data are means of five replications of four eggs each that measured at the end of each period.

**TABLE 6:** Effect of diet digestible lysine (dLys) concentration during 11–22 weeks of age on egg composition, egg fertility and hatchability and new‐born‐chick live body weight of breeding Japanese quail determined at the end of the experiment.

	Egg composition								Reproductive performance			
	Whole egg without shell, %			Yolk, %			Albumen, %			Hatchability, %		

Analysed dLys concentration, %	Solids	Ether extract	Crude protein	Solids	Ether extract	Crude protein	Solids	Crude protein	Fertility, %	Set eggs	Fertile eggs	Chick live weight
0.690	25.33	10.68	14.93	49.76	31.81	18.19	13.00	13.28	93.96	88.75	94.32	9.07
0.755	25.97	10.97	15.10	51.40	32.73	18.77	13.24	13.25	90.88	86.50	95.16	9.09
0.820	25.31	10.86	14.33	51.68	32.63	18.08	12.18	12.48	89.69	85.32	95.40	9.14
0.885	24.95	10.68	15.04	49.16	32.49	18.62	13.07	13.31	94.62	88.69	93.95	8.97
0.950	24.74	10.75	14.97	49.70	33.22	18.86	12.89	13.12	89.80	85.30	94.90	8.78
1.015	26.06	11.34	14.82	49.10	30.97	18.37	12.62	12.78	93.56	84.57	90.43	8.96
SEM	0.606	0.680	0.327	0.906	1.523	0.441	0.380	0.388	2.321	2.702	2.226	0.334
Dietary dLys levels response, *p*‐value												
Linear	0.392	0.754	0.750	0.303	0.393	0.655	0.811	0.836	0.386	0.987	0.296	0.849
Quadratic	0.393	0.735	0.751	0.269	0.386	0.673	0.836	0.861	0.385	0.973	0.267	0.790

*Note*: Data are means of 5 replications of pooled 10 samples for egg composition and 50 egg samples that incubated for reproductive performance traits.

### 3.5 Fertility and hatchability

The effects of dietary l‐Lys‐HCl supplementation on reproductive attributes, including fertility and hatchability (as hatched chicks from total egg sets), are presented in Table 6. The supplementation of the diet with all tested levels of l‐Lys‐HCl did not cause significant changes on the studied parameters (*p* > 0.05).

### 3.6 Blood metabolites and immune response

The effects of dietary l‐Lys‐HCl supplementation on blood metabolites and the immune response to SRBC inoculation are presented in Tables 7 and 8, respectively. The supplementation of the diet with different levels of l‐Lys‐HCl did not affect (*p *> 0.05) blood metabolites except AU and ALP concentration which had a significant desire (0.05 < *p* < 0.10). Dietary supplementation of l‐Lys did not affect the primary and secondary immune responses to SRBC inoculation (*p* > 0.05).

**TABLE 7:** Effect of diet digestible lysine (dLys) concentration during 11–22 weeks of age on blood metabolites of breeding Japanese quail determined at the end of the experiment.

Analysed dLys concentration, %	TG, mg/dL	Cho, mg/dL	HDL, mg/dL	ALT, U/L	AST, U/L	ALP, U/L	TP, g/dL	Cr, mg/dL	UA, mg/dL
0.690	86	196	102	6.00	222	2606	4.60	0.58	7.78
0.755	85	255	98	6.50	242	1930	4.95	0.28	8.20
0.820	107	223	99	4.75	235	1643	4.48	0.58	8.10
0.885	88	234	95	6.00	197	1897	4.65	0.55	8.60
0.950	92	218	106	5.75	253	1992	4.60	0.58	8.58
1.015	97	212	96	5.75	222	1843	4.55	0.50	7.53
SEM	4.699	17.701	4.979	0.899	17.014	207	0.253	0.058	0.358
Dietary dLys levels response, *p*‐value									
Linear	0.435	0.197	0.819	0.710	0.987	0.072	0.933	0.994	0.083
Quadratic	0.468	0.196	0.832	0.720	0.985	0.084	0.913	0.975	0.083

*Note*: Data are means of five replications.

Abbreviations: ALP, alkaline phosphatase; ALT, alanine aminotransferase; AST, aspartate aminotransferase; Cho, cholesterol; Cr, creatinine; TG, triglyceride; TP, total protein; UA, uric acid.

**TABLE 8:** Effect of diet digestible lysine (dLys) concentration during 11–22 weeks of age on antibody titres responses to sheep red blood cell (SRBC) inoculation responses of breeding Japanese quail determined at the end of the experiment.

	Antibody concentration responses to SRBC inoculation at 18 and 19 weeks of age (log_2_)					
Analysed dLys concentration, %	7 days after the first injection			7 days after the second injection		
	Total antibody	IgY	IgM	Total antibody	IgY	IgM
0.690	4.40	2.80	1.60	6.20	3.60	2.60
0.755	4.60	2.40	2.20	6.00	3.80	2.20
0.820	4.80	2.60	2.20	6.60	4.20	2.40
0.885	4.60	2.40	2.20	5.80	3.40	2.40
0.950	5.00	2.80	2.20	6.00	4.20	1.80
1.015	4.40	2.40	2.00	5.80	3.80	2.00
SEM	0.197	0.337	0.357	0.387	0.329	0.253
Dietary dLys levels response, *p*‐value						
Linear	0.103	0.799	0.231	0.722	0.642	0.936
Quadratic	0.109	0.815	0.244	0.686	0.659	0.981

*Note*: The values are means of five replicates. Data represent means of log_2_ of the reciprocal of the last dilution exhibiting agglutination.

### 3.7 Estimated digestible lysine requirement

**FIGURE 1:**
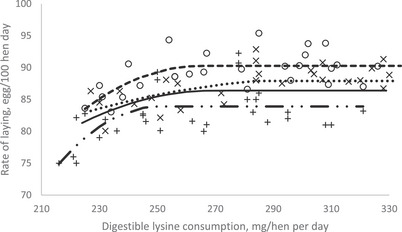
Plot of hen‐day egg production (*Y*, in %) vs. daily digestible lysine consumption (*X*, in mg/bird/day) of breeding Japanese quail during periods (×) 11–14, (○) 15–18 and (+) 19–22 weeks of age of the first laying cycle. (…) Quadratic broken‐line fitted model for 11–14 weeks of age period; *Y* = 87.34 − 0.001 (272 − *X*)^2^ × I, *I* = 1 (if *X* < 272 or *I* = 0 (if *X* > 272), *p* < 0.003, adj. *R* ^2^ = 0.34; the break point occurred at 272 ± 35.75. (—‐) Quadratic broken‐line fitted model for 15–18 weeks of age period; *Y* = 90.26 − 0.004 (265 − *X*)^2^ × *I*, *I* = 1 (if *X* < 265 or *I* = 0 (if *X* > 265), *p* < 0.001, adj. *R* ^2^ = 0.40; the break point occurred at 265 ± 16.39. (–..–) Quadratic broken‐line fitted model for 19–22 weeks of age period; *Y* = 83.90 − 0.008 (250 − *X*)^2^ × *I*, *I* = 1 (if *X* < 250 or *I* = 0 (if *X* > 250), *p* < 0.003, adj. *R* ^2^ = 0.35; the break point occurred at 250 ± 15.93. (—) Quadratic broken‐line fitted model for overall experimental period (11–22 weeks of age); *Y* = 86.39−0.003 (266 − *X*)^2^ × *I*, *I* = 1 (if *X* < 266 or *I* = 0 (if *X* > 266), *p* < 0.027, adj. *R* ^2^ = 0.24; the break point occurred at 266 ± 25.33.

**FIGURE 2:**
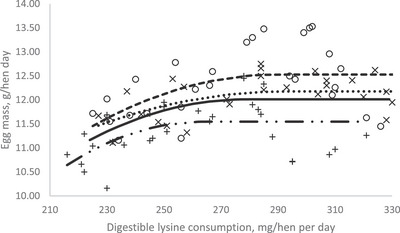
Plot of egg mass (*Y*, in g/bird/day) vs. daily digestible lysine consumption (*X*, in mg/bird/day) of breeding Japanese quail during periods (×) 11–14, (○) 15–18 and (+) 19–22 weeks of age of the first laying cycle. (…) Quadratic broken‐line fitted model for 11–14 weeks of age period; *Y* = 12.18 − 0.0002 (293 − *X*)^2^ × *I*, *I* = 1 (if *X* < 293 or *I* = 0 (if *X* > 293), *p* < 0.017, adj. *R* ^2^ = 0.12; the break point occurred at 293 ± 36.09. (—) Quadratic broken‐line fitted model for 15–18 weeks of age period; *Y* = 12.53 − 0.0003 (285 − *X*)^2^ × *I*, *I* = 1 (if *X* < 285 or *I* = 0 (if *X* > 285), *p* < 0.034, adj. *R* ^2^ = 0.21; the break point occurred at 285 ± 38.57. (–..–) Quadratic broken‐line fitted model for 19–22 weeks of age period; *Y* = 11.54 − 0.0004 (264 − *X*)^2^ × *I*, *I* = 1 (if *X* < 264 or *I* = 0 (if *X* > 264), *p* < 0.036, adj. *R* ^2^ = 0.19; the break point occurred at 264 ± 27.92. (—) Quadratic broken‐line fitted model for overall experimental period (11–22 weeks of age); *Y* = 12.01 − 0.0003 (275 − *X*)^2^ × *I*, *I* = 1 (if *X* < 275 or *I* = 0 (if *X* > 275), *p* < 0.021, adj. *R* ^2^ = 0.25; the break point occurred at 275 ± 30.16.

**FIGURE 3:**
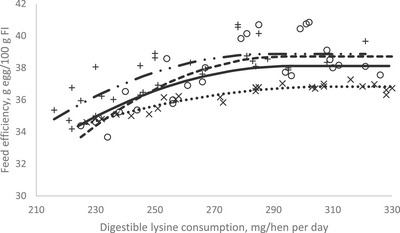
Plot of feed efficiency (*Y*, in g egg mass/g feed intake) vs. daily digestible lysine consumption (*X*, in mg/bird/day) of breeding Japanese quail during periods (×) 11–14, (○) 15–18 and (+) 19–22 weeks of age of the first laying cycle. (…) Quadratic broken‐line fitted model for 11–14 weeks of age period; *Y* = 36.82 − 0.0003 (303 − *X*)^2^ × *I*, *I* = 1 (if *X* < 303 or *I* = 0 (if *X* > 303), *p* < 0.001, adj. *R* ^2^ = 0.91; the break point occurred at 303 ± 8.85. (—) Quadratic broken‐line fitted model for 15–18 weeks of age period; *Y* = 38.71 − 0.001 (294 − *X*)^2^ × *I*, *I* = 1 (if *X* < 294 or *I* = 0 (if *X* > 294), *p* < 0.001, adj. *R* ^2^ = 0.64; the break point occurred at 294 ± 16.22. (–..–) Quadratic broken‐line fitted model for 19–22 weeks of age period; *Y* = 38.87 − 0.001 (281 − *X*)^2^ × *I*, *I* = 1 (if *X* < 281 or *I* = 0 (if *X* > 281), *p* < 0.001, adj. *R* ^2^ = 0.65; the break point occurred at 281 ± 18.11. (—) Quadratic broken‐line fitted model for overall experimental period (11–22 weeks of age); *Y* = 38.12 − 0.0007 (293 − *X*)^2^ × *I*, *I* = 1 (if *X* < 293 or *I* = 0 (if *X* > 293), *p* < 0.001, adj. *R* ^2^ = 0.83; the break point occurred at 293 ± 11.03.

In order to investigate the effect of dietary dLys level on productive and reproductive performance, egg quality, blood metabolites and immune response in breeding Japanese quails during the first production phase, a crucial goal with this study was to estimate their dLys requirement. The optimization model was solved using the NLIN program SAS 9.1 procedure. Fitted broken‐line models for the egg performance traits during 11–14, 15–18, 19–22 and 11–21 (overall) weeks of age periods as a function of daily dLys consumption are shown in Table 9 and Figures 1, 2, 3. The dLys requirements for optimized EP during the 11–14, 15–18, 19–22 and 11–22 (overall) weeks of age periods were estimated at 272, 265, 250 and 266 mg/bird/day, respectively (Table 9 and Figure 1). The dLys requirements for the optimal EM were estimated at 293, 285, 264 and 279 mg/hen/day during the 11–14, 15–18, 19–22 and 11–22 (overall) weeks of age, respectively (Table 9 and Figure 2). The dLys requirements for the optimal FE were estimated for 11–14, 15–18, 19–22 and 11–22 (overall) weeks of age at the amounts of 303, 294, 281 and 293 mg/hen/day, respectively (Table 9 and Figure 3). The optimum values estimated for FE were higher compared to those estimated for EM and EP. The egg quality and reproductive traits did not fit in the models to estimate the dLys requirements.

**TABLE 9:** Estimated digestible lysine (dLys) requirements (mg/bird/day) of breeding Japanese quail during periods 11–14, 15–18 and 19–22 weeks of age and the whole experimental period (overall: 11–22 weeks) for optimization of egg production parameters by quadratic broken‐line regression fit models.^a^

Parameters	Estimated requirement ^b^	Approximate confidence limits		*p*‐Value	Adj. *R* ^2^	Predicted value ^c^	Equation ^d^
		Lower 95%	Upper 95%				
Egg production, egg/100 hen day							
11–14 weeks	272	234	310	0.003	0.34	87.34	*Y* = 87.34 − 0.001(*R* − *X*)^2^ × *I*
15–18 weeks	265	231	298	0.001	0.40	90.26	*Y* = 90.26 − 0.004(*R* − *X*)^2^ × *I*
19–22 weeks	250	218	283	0.003	0.35	83.90	*Y* = 83.90 − 0.008(*R* − *X*)^2^ × *I*
Overall (11–22 weeks)	266	217	315	0.027	0.24	86.36	*Y* = 86.39 − 0.003(*R *– *X*)^2^ × *I*
Egg mass, g egg/hen day							
11–14 weeks	293	217	367	0.017	0.12	12.18	*Y* = 12.18 − 0.00016(*R* − *X*)^2^ × *I*
15–18 weeks	285	210	364	0.034	0.21	12.53	*Y* = 12.53 − 0.00026(*R* − *X*)^2^ × *I*
19–22 weeks	264	206	320	0.036	0.19	11.54	*Y* = 11.54 − 0.0004(*R* − *X*)^2^ × *I*
Overall (11–22 weeks)	275	217	333	0.021	0.25	12.01	*Y* = 12.01 − 0.00028(*R* − *X*)^2^ × *I*
Feed efficiency (g EM/100 g FI)							
11–14 weeks	303	291	327	0.001	0.91	36.82	*Y* = 36.82 − 0.00034(*R* − *X*)^2^ × *I*
15–18 weeks	294	260	327	0.001	0.64	38.71	*Y* = 38.71 − 0.0011(*R* − *X*)^2^ × *I*
19–22 weeks	281	251	325	0.001	0.65	38.87	*Y* = 38.87 − 0.0008(*R* − *X*)^2^ × *I*
Overall (11–22 weeks)	293	274	312	0.001	0.83	38.11	*Y* = 38.12 − 0.0007(*R* − *X*)^2^ × *I*

*Note*: *R*, estimated requirement; Adj. *R* ^2^, adjusted coefficient of determination.

Abbreviations: EM, egg mass; FI, feed intake.

^a^ Responses did not fit in the models to estimate the dLys requirements for egg weight and feed intake.

^b^ Expressed as mg/bird/day.

^c^ Predicted values by models to obtain optimum egg production (%); optimum egg mass (g/bird/day) and optimum feed conversion ratio.

^d^ *I* = 1 (if *X* < *R* or *I* = 0 (if *X* > *R*).

## 4 DISCUSSION

### 4.1 Feed and nutrient intake

In the current experiment, the breeding quails had an average daily FI of 32.49 g/bird, and corresponding to that, ME consumption was 95 kcal/bird/day and protein consumption was 5.36 g/bird/day. All of these amounts are higher than laying Japanese quail requirements (FI, 24.8–26.5 g; ME, 74 kcal; and protein, 4.94 g/bird/day) as recommended in the Brazilian Tables for Poultry and Swine (Rostagno et al., 2011). The probable reason for the higher ME and protein intake of breeding quails in the current experiment as compared to the lower amount reported by Brazilian Tables for Poultry and Swine is the higher LBW of birds used in the current experiment (348 ± 8.40 g/bird LBW vs. 165–190 g/bird LBW). The strain of quails used in the current experiment was selected for meat and egg yields. This selection procedure resulted in a slightly heavier LBW with higher EW (average EW was 13.65 ± 0.33 g). Another difference is that in the current experiment, the breeding quails had reached production peak and adult body weight. This achievement is in accordance with Lima et al. (2016) who reported that heavier quails showed higher EM/bird/day.

In the current study, the diet dLys levels did not affect FI. The current results are similar to results reported by previous researchers, who reported that FI of the quils was not affected by dietary dLys concentrations (Garcia et al., 2005; Lima et al., 2016; Pinto et al., 2003; Ribeiro et al., 2003, 2013). Similarly, results from laying hens showed that feed consumption was not affected by supplemental Lys (Novak et al., 2004; Prochaska et al., 1996; Schutte & Smink, 1998). In contrast, some reports indicated that feed consumption in the Japanese quails during the laying phase was affected by supplemental Lys with quadratic (Rodrigues et al., 2007) or linear (Costa et al., 2008) trends. In the current study, a linear increase (*p* < 0.001) in dLys intake was verified as its concentration in the diet increased. These results are similar to those reported by Ribeiro et al. (2013) and Rodrigues et al. (2007), who found a linear increase in Lys intake from increasing dLys/kg of diet. Non‐significant variations were seen in almost all other nutrients and ME consumption.

### 4.2 Live body weight change

The average LBW of the birds used in the current experiment was 348 ± 8.40 g/bird. The strain of quails used in the current experiment was selected for meat and egg yields. This selection procedure resulted in a slightly heavier LBW. During the experiment, birds LBW change was not significantly affected by dietary Lys levels. Pinto et al. (2003) observed that the body weight of female Japanese quail at 23 weeks of age was not significantly affected by feeding diets with varying levels of 0.8%–1.3% dLys. However, it was reported that body weight gain in laying hens was affected by Lys intake during 20–60 weeks of age (Novak et al., 2004).

### 4.3 Productive performance

The results obtained from this experiment showed that the productive performance traits were improved by a quadratic polynomial trend during the first, second, third and whole experimental periods in response to increasing dLys levels in the diet. The highest EP, EM and FE were observed in birds that fed with a diet containing 0.885% dLys. Our results in the current study are similar to those found by previous researchers, who reported an improvement in laying quail productive performance following the increase in dietary Lys supplementation (Lima et al., 2016; Pinto et al., 2003; Shim & Lee, 1984). Shim and Lee (1984) reported that feed conversion ratio and EP were improved with 0.97% of Lys in the diet. This improvement in performance indicators by increasing levels of Lys in the diet can provide higher percentages of protein deposits (Tatiane da Silva et al., 2021). The level of a single essential amino acid that is either deficient or in excess may result in a diet that does not optimize the economic efficiency of a laying birds’ production system (Hossaninejad et al., 2021; Lima et al., 2016). In our study, by increasing dietary dLys concentration, to higher levels (up to 0.95% and 1.015%), productive performance traits became unfavourable. This result is consistent with the previous research which reported a quadratic effect (*p* < 0.01) of dLys levels on EP (Costa et al., 2008; Nery et al., 2015) and EM (Pinto et al., 2003; Ribeiro et al., 2013; Robbins et al., 2006), and or feed conversion ratio was poorer at the higher (1.375%) Lys levels (Ribeiro et al., 2013). Similarly, results on laying hens revealed a quadratic effect of dietary dLys levels on EP (Akbari Moghaddam Kakhki et al., 2016), and feed conversion ratio (Martinze et al., 2005).

### 4.4 Egg weight, quality and composition

In the current experiment, the average EW and relative albumen, yolk and shell weight were not affected (*p* > 0.05) by the dLys levels in the diets. This result is in agreement with those did not verify changes in Japanese quail's EW (Garcia et al., 2005; Rodrigues et al., 2007), albumen, yolk and eggshell relative weight (Costa et al., 2008), and laying hens EW during production peak (Schutte & Smink, 1998) resulting from the increase in dietary Lys levels. In contrast, some researchers reported improvements in breeding Japanese quails (Pinto et al., 2003; Ribeiro et al., 2013; Shim & Lee, 1985), laying hens (Akbari Moghaddam Kakhki et al., 2016) and laying ducks (Fouad et al., 2018) egg size by increasing Lys levels. Ribeiro et al. (2013) reported that diet dLys levels showed a quadratic effect (*p* < 0.05) on laying quail EW. The 11.20 g/kg diet dLys concentration promoted the highest EW. Moreover, yolk and albumen weights were significantly increased in comparison with the group that consumed a diet containing 0.95% Lys. However, Pinto et al. (2003) reported a significant reduction in eggshell relative weight by increasing diet Lys levels from 0.890% to 1.300%. The average EW, albumen, yolk and shell yield of eggs in the current experiment were 13.65 ± 0.33 (g/egg), 61.23 ± 1.86, 30.39 ± 1.78 and 8.38 ± 0.34 (g/100 g egg), respectively.

In any or the whole experimental period, the egg quality traits, such as egg shape index, Haugh unit, egg SG and shell thickness, were not significantly affected by the dietary dLys levels. Overall, the averages of egg shape index, Haugh unit, egg SG and shell thickness were 77.50 ± 1.74, 85.75 ± 2.50, 1.066 ± 0.004 (g/cm^3^) and 258 ± 14.6 (µm), respectively. In agreement with our result in the current experiment, increasing dietary dLys levels from 0.657% to 0.857% in Hy‐Line W36 laying hens did not affect egg shape index (Akbari Moghaddam Kakhki et al., 2016) and eggshell thickness (Hossaninejad et al., 2021; Kumari et al., 2016).

In accordance with Applegate et al. (2009), dry matter and protein contents of yolk, albumen and the whole egg liquid were not influenced by dietary dLys concentration. But our observation in the current experiment was inconsistent with some authors (Novak et al., 2004; Prochaska et al., 1996), who worked on laying hens. The average DM of yolk, albumen and liquid egg (total egg without shell) in our experiment were 50.13 ± 2.27, 12.83 ± 0.92 and 25.39 ± 1.44 g/100 g, respectively. The average protein content in the yolk, albumen and liquid egg were 18.48 ± 1.02, 13.04 ± 0.92 and 14.87 ± 0.77 g/100 g DM, respectively.

### 4.5 Fertility and hatchability

In the current study, the reproductive performance traits, such as fertility and hatchability, were not significantly affected by dietary Lys supplementation. In contrast, it has been reported that increasing the level of Lys from 0.9% to 1.3% of the diet improves egg fertility and hatchability (Shim & Lee, 1985). Few studies have been conducted to evaluate the effects of dietary Lys supplementation on breeding quails’ reproductive performance. Therefore, it is very difficult to compare our findings with others.

### 4.6 Blood metabolites

Data presented in Table 7 indicate that blood serum UA and ALP concentrations were significantly affected by increasing dietary dLys levels. However, other blood metabolites were not affected by diet dLys concentration. Blood serum ALP and UA concentrations were the lowest in the birds fed with diet containing 0.82% dLys. In contrast, no significant effect on plasma UA concentration was observed by increasing the level of Lys in the diet of laying hens (Akbari Moghaddam Kakhki et al., 2016).

### 4.7 Immune response

The HA test showed that there was no antibody against SRBC in the sera collected from the SRBC non‐inoculated birds. In the current study, dietary Lys supplementation did not significantly affect the antibody titres to SRBC in quails. In contrast to our observations, serum antibody titres were progressively and significantly increased as the dietary concentrations of Lys increased up to 11.41 g/kg at both 0.5% and 2.5% SRBC concentrations (Praharaj et al., 2002). Further increase in Lys level did not elicit any positive response in producing more antibodies.

### 4.8 Estimated digestible lysine requirement

The best balance of performance traits and daily dLys consumption were estimated at the 272, 293 and 303 mg/bird/day during the 11–14 weeks of age for EP, EM and FE, respectively. This value was obtained lower (265, 285 and 294 mg/bird/day) during 15–18 weeks of age and lower (250, 264 and 281 mg/bird/day) during 19–22 weeks of age. Overall, the predicted dLys requirements throughout the whole experiment (11–22 weeks of age) for optimized EP, EM and FE were estimated at 266, 275 and 293 mg/bird/day, respectively. The Brazilian Tables for Poultry and Swine (Rostagno et al., 2011) recommended dLys requirements of laying Japanese quails vary according to laying performance from 268 to 288 mg/bird/day. Furthermore, previous studies estimated dLys requirements of quails during laying phase at 292 (Costa et al., 2008), 291 (Carvalho et al., 2022), 275 (Garcia et al., 2005), 272 (Ribeiro et al., 2003) and 254 (Pinto et al., 2003) mg/bird/day.

Corresponding to average daily feed consumption, 32.49 ± 1.05 g/bird/day, the current achievement suggests that breeding Japanese quail diet containing 0.932%, 0.905%, 0.865% and 0.902% dLys concentration is marginally adequate during the 11–14, 15–18 and 19–22 weeks of age and the whole experiment (11–22 weeks of age). Several authors performed studies evaluating the introduction of dLys for EP in Japanese quails verified the optimal level with the supply of 1.18% (Lima et al., 2016), 1.117% (Pinto et al., 2003), 1.08% (Nery et al., 2015), 1.03% (Costa et al., 2008) and 0.90% (Tatiane da Silva et al., 2021) dietary dLys concentrations. The dietary dLys level recommendations for Japanese quails in the Brazilian Tables for Poultry and Swine vary according to laying performance and FI from 1.045% to 1.097% (Rostagno et al., 2011).

The current study achievement showed that the estimation of dLys requirement depends on what parameter is taken into consideration for optimization. The dLys requirements to optimise the FE were higher compared to those for EM and EP (293 vs. 275 and 266 mg/bird/day for whole experimental period). It is well known that Lys supplementation improves amino acid balance and, consequently, promotes EP by increasing protein synthesis and decreasing fat synthesis, leading to enhanced FE. Regarding the literature, there are contradictory results in the estimation of Lys requirements for laying Japanese Quails. This variation may be explained by differences between present quails. These birds performed differently because of the management practices and genetic selection (Reda et al., 2020). Moreover, maybe the experiments were conducted in different environmental conditions, basal diets with variations in feed ingredients and dietary energy and nutrient concentration, bird's age, estimation methods and traits that are taken into consideration for optimization. All of them are reasons for inconsistent results in the estimations (Pontin et al., 2018; Robbins et al., 2006; Severo et al., 2019). Therefore, upgrading the nutritional supplies of poultry might be an essential strategy in commercial rearing systems (Reda et al., 2020).

## 5 CONCLUSION

The outcomes of the current examination revealed that Lys is indispensable amino acid in breeding Japanese quails fed corn‐soybean meal diet during the first production cycle. Estimation of dLys requirement depended on EP phase and traits that are taken considered for optimization. The dLys requirement estimated for FE was higher than those for EP and EM. During the peak stage of first laying cycle, the dietary dLys level of 0.932% and a daily intake of 303 mg dLys per bird are adequate for optimized performance.

## AUTHOR CONTRIBUTIONS

Mohammad Amin Omary out the experimental trail lab analysis. Heydar Zarghi designed the experimental trail, performed the statistics, tabulated the data, wrote the draft and reviewed the manuscript. Ahmad Hassanabadi reviewed the manuscript.

## CONFLICT OF INTEREST STATEMENT

The authors declare no conflicts of interest.

### ETHICS STATEMENT

The authors confirm that the ethical policies of the journal, as noted in the journal's author guidelines page, have been adhered to and the appropriate ethical review committee approval has been received (IR.UM.REC.1402.149). The authors confirm that they have followed EU standards for protecting of animals used for scientific purposes and feed legislation.

### PEER REVIEW

The peer review history for this article is available at https://publons.com/publon/10.1002/vms3.70038.

## DECLARATIONS

The authors declare that all of the authors listed on the manuscript employed at an academic or research institution where research or education is the primary function of the entity. Moreover, this manuscript is independently submitted by the authors.

## Data Availability

The data that support the findings of this study are included in this published article.
